# Non-invasive assessment of exfoliated kidney cells extracted from urine using multispectral autofluorescence features

**DOI:** 10.1038/s41598-021-89758-4

**Published:** 2021-05-20

**Authors:** Saabah B. Mahbub, Long T. Nguyen, Abbas Habibalahi, Jared M. Campbell, Ayad G. Anwer, Uzair M. Qadri, Anthony Gill, Angela Chou, Muh Geot Wong, Martin E. Gosnell, Carol A. Pollock, Sonia Saad, Ewa M. Goldys

**Affiliations:** 1 ARC Centre of Excellence for Nanoscale Biophotonics, UNSW Sydney, Sydney, NSW 2052 Australia; 2 Graduate School of Biomedical Engineering, UNSW Sydney, Sydney, NSW 2052 Australia; 3 School of Mathematical and Physical Sciences, Faculty of Science, University of Technology, Sydney, Australia; 4 Kolling Institute of Medical Research, Royal North Shore Hospital, St Leonards, NSW 2065 Australia; 5 NSW Health Pathology, Department of Anatomical Pathology, Royal North Shore Hospital, Sydney, NSW 2065 Australia; 6 Sydney Medical School, University of Sydney, Sydney, NSW 2006 Australia; 7 The George Institute for Global Health, Sydney, NSW 2050 Australia; 8 Quantitative Pty Ltd, 118 Great Western Highway, Mount Victoria, NSW 2786 Australia

**Keywords:** Computational biophysics, Machine learning, Chronic kidney disease

## Abstract

Optimally preserved urinary exfoliated renal proximal tubule cells were assessed by multispectral imaging of cell autofluorescence. We demonstrated different multispectral autofluorescence signals in such cells extracted from the urine of patients with healthy or diseased kidneys. Using up to 10 features, we were able to differentiate cells from individuals with heathy kidneys and impaired renal function (indicated by estimated glomerular filtration rate (eGFR) values) with the receiver operating characteristic area under the curve (AUC) of 0.99. Using the same method, we were also able to discriminate such urine cells from patients with and without renal fibrosis on biopsy, where significant differences in multispectral autofluorescence signals (AUC = 0.90) were demonstrated between healthy and diseased patients (*p* < 0.05). These findings show that multispectral assessment of the cell autofluorescence in urine exfoliated proximal tubule kidney cells has the potential to be developed as a sensitive, non-invasive diagnostic method for CKD.

## Introduction

Non-invasive molecular level assessment of deep organs such as kidneys remains a significant challenge. Standard magnetic resonance imaging (MRI), computed tomography (CT) scans and positron emission tomography (PET) and single photon positron emission tomography (SPECT) are principally limited to the detection of masses, density changes or morphology/texture with findings being impacted by poor image quality, motion artifacts or reliance on contrast agents^1–3^.

Multiple cells are exfoliated from the kidney into the urine daily^4^. These include podocytes, progenitor cells and proximal tubule epithelial cells (PTCs)^5^. The proximal tubules make up the majority of the kidney mass. Proximal tubule-related pathology is a hallmark of multiple kidney diseases and it correlates with future decline in kidney function^6,7^. This suggests that the biology of these non- invasively obtained cells could give a direct window into kidney pathology. Indeed, it has been established that a small number of exfoliated PTCs can be isolated and cultured on collagen plates for signal amplification^8,9^. The disadvantage of this approach is the loss of cellular properties during culture that could be pivotal for diagnosis. The currently available clinical methods to identify CKD are insufficient because the physiological markers used currently only become abnormal once significant pathology is evident. Hence non-invasive methods of diagnosing CKD are required and examination of cells exfoliated from the kidney using novel isolation and assessment techniques presents an attractive opportunity.

Cell autofluorescence originates from native fluorophores such as collagen, elastin, tryptophan and reduced nicotinamide adenine dinucleotide (phosphate) (NAD(P)H), and flavins^10,11^, which play a pivotal role in cell and tissue metabolism. Multispectral microscopy can be used to collect native emission data across a broad range of excitation wavelengths. This data yields the spectral profile of autofluorescent features for each cell, including parameters such as average channel intensities, channel intensity ratio^12^, pixel standard deviations and skewness^13^. This gives each cell a unique signature, arising directly from its intracellular biochemistry and organisation, which has been used in the literature for the sensitive discrimination of specific cellular characteristics, which have included cell cycle stage^14^, the presence of inflammatory disease^15^, levels of reactive oxygen species^16^, neoplasia^10,17^ and age^18^. We have previously demonstrated increased oxidative stress markers in the proximal tubules of animals with early diabetic or obesity related kidney disease^19,20^. Oxidative stress is a key factor affecting kidney function^21,22^. Multispectral assessment of cell autofluorescence has been specifically shown to be sensitive to metabolic changes and oxidative stress^14,16^. As such, it could be expected that the application of multispectral autofluorescence microscopy to exfoliated renal tubule cells will enable the sensitive, non-invasive characterisation of kidney dysfunction, providing a new window into physiology of this deep organ.

In this work, we demonstrate that multispectral autofluorescence colour and colour-dependent morphology measured in a small number of exfoliated kidney cells (PTCs) reflects established markers of kidney disease determined by assessing estimated glomerular filtration rate (eGFR) and renal pathology (current gold standard measure obtained via invasive kidney biopsy)^23^. We also present an optimised protocol which enables the preservation and characterisation of exfoliated proximal tubule cells without the requirement of cell culture.

## Materials and methods

### Patients characteristics

Participants of either sex aged between 18 and 75 years of age were included and informed consent was obtained from all participants involved in this study. The study was carried out in accordance with relevant guidelines and regulations was approved by the human Ethic committee at Royal North Shore Hospital (Ref: HREC/17/HAWKE/471) and University of New South Wells, Sydney (Ref: HC180710). Two cohorts of patients were assessed in this study. The first cohort included 22 patients with Type 2 diabetes with varying levels of eGFR. Patients were grouped as follows: Group 1 (preserved kidney function with eGFR > 60 ml/min/1.73 m^2^), Group 2—(impaired kidney function with eGFR < 60 ml/min/1.73 m^2^. Patients were included if they had a diagnosis of type 2 diabetes and had been receiving anti-diabetic treatment (diet controlled, oral hypoglycaemics and/or insulin) for at least 12 months prior to the procedure.

The second cohort included 10 patients undergoing nephrectomy or clinically indicated kidney biopsy. Renal tissue was assessed for objective markers of tubulointerstitial fibrosis in haematoxylin, Periodic Acid Schiff and Masson’s trichrome stained paraffin embedded tissue as per routine practice using semi- quantitative scores for each of the following parameters: tubular atrophy, interstitial fibrosis, arteriolar hyalinosis, interstitial inflammation and arterial intimal fibrosis. The patients were grouped as follows: Group 1—Control with no renal interstitial fibrosis and tubular atrophy (IFTA) and Group 2—with detectable IFTA in the kidneys. Kidney pathology was assessed in a blinded manner by 2 different pathologists as we have previously described^19,24^. Healthy tissues with no detectable IFTA were obtained from patients undergoing a nephrectomy generally undertaken for tumour resection, with the tissue analysed being remote from the tumour. Urine was collected for proximal tubule cells extraction prior to the nephrectomy or biopsy. Details of patient groups from the first and second cohort are provided in Supplementary Table 1a, b.

### Extraction of proximal tubule cells

Urine samples (100 ml) were collected from patients fulfilling the above criteria. The urine was placed on ice immediately after collection for transportation, then spun at 4℃ before freezing it down and stored frozen prior to assessment. Renal proximal tubular cells were extracted from the thawed urine using immune- magnetic separation, anti-CD13 and anti-Sodium-glucose linked transporter-2 (SGLT2) antibodies. Briefly, urine cells were spun at 3000/4000 rpm for 20 min (at 4 °C) and then washed twice with phosphate buffered-saline (PBS). Cells were stored at 4 °C overnight or − 80 °C in Dulbecco's Modified Eagle Medium (DMEM), Hanks' Balanced Salt solution (HBSS) or Phosphate buffer saline (PBS) with 10% Dimethyl Sulfoxide (DMSO) to assess the optimal storage condition and maximal cell viability. On the day of the experiment, frozen cells were quickly thawed at 37 °C and then washed in PBS. Renal proximal tubule cells were then extracted following incubation with 5 µg of mouse anti-human CD13 antibody (Invitrogen) and 2.5 µg of SGLT2 antibody (Abcam) for 1 h (at 4 °C) followed by 5 µl of CELLection Pan anti-mouse dynabeads (Invitrogen) for 30 min (at 4 °C). Specific proximal tubule cells bound to a magnet were then washed in PBS before elution from the beads according to the manufacturer’s instructions. The number of exfoliated proximal tubule cells in 100 ml urine is around 20–50 cells as previously demonstrated^25^. These cells were resuspended in 50 μl of PBS on a 35 mm glass bottom petri dish (Cell E&G, USA, and # GDB0004-200) then assessed using a multispectral and brightfield microscopy. Due to field restriction, only a fraction of the total number of cells per sample can be imaged.

### Flow cytometry

Cell viability was assessed by flow cytometry (LSRFortessa, BD Bioscience, MA, USA) using either 7- Aminoactinomycin D (7-AAD) or propidium iodide (PI) staining as previously described^26^. Briefly, urine cells were collected as described above and washed twice with PBS before incubation with either 5 μl of 7-AAD (BD Bioscience, MA, USA) or 5–10 μl of PI (Invitrogen, CA, USA) for 10 min at room temperature in the dark. Human kidney (HK2) cells were used as a positive control. In a separate experiment, urinary exfoliated cells were collected, and cells were incubated with 5 μl of PE-conjugated anti-CD13 antibody (Invitrogen, CA, USA), an epithelial marker, and 2 μl of FITC conjugated anti-SGLT2 antibody (Abcam, Cambridge, UK), a proximal tubule marker, for 1 h at room temperature in the dark. Cells were then washed and resuspended in PBS and subjected to the flow cytometer for fluorescence analysis. The population has been gated to exclude the debris and dead cells. We also added EDTA to the washing buffer to dissolve precipitates.

### Confocal microscopy

Similar to flow cytometry, urinary exfoliated cells were incubated with CD13, SGLT2 and angiotensinogen (AGT) at 4 °C overnight, washed with PBS and stained with fluorophore-conjugated secondary antibodies when necessary. The cells were washed twice with ice cold PBS, stained with 1 drop of nuclear dye (Hoechst 33342, Invitrogen, CA, USA) and loaded onto 35 mm coverslip-bottom dishes for imaging. Fluorescent signals before and after proximal tubular cell enrichment was assessed using a confocal microscope (Olympus FV3000, Shinjuku, Tokyo, Japan).

### Multispectral and brightfield imaging system

A custom-made autofluorescence microscopy system^11,12,27^ was built by adapting a standard fluorescence microscope (Olympus iX83™). Multispectral excitation lamp (from Quantative™, AU) has been applied to excite the single photon-excited autofluorescence signal of cell samples in a number of defined narrowband (± 5 nm) excitation wavelength ranges provided by low power LEDs and several epifluorescence filter cubes to produce defined spectral channels (34 channels). The channels span wide excitation (340–510 nm) and emission (420–650 nm) wavelength ranges (channel details are given in Supplementary Table 1). For imaging we used a 40× water (NA 1.15) objective, with transmission in the UV range. All images were captured by Photometrics Prime95B™ sCMOS camera (Sensor: GPixel GSense 144 BSI CMOS Gen IV, Teledyne Photometrics, AZ, US) operated below − 30 °C to reduce sensor-induced noise. The image size of the camera is 1200 × 1200 pixels. We used image acquisition times of up to 5 s per channel, with multiple averaging (3 times) to optimise image quality (i.e. SNR). A sequence of fluorescence images was performed of the same sample area in each of these spectral channels (collectively named a “data block”). Each data block is supplemented by a brightfield image of the sample area which is used as a broad reference. Representative bright field and spectral channel cell images from each group are presented in Supplementary Fig. 3 l. Imaging of the native fluorescence emission of the cell samples and reference images were carried out: dark, water, and calibration fluid (30 µM NADH mixed with 18 µM FAD) in multiple distinctive spectral channels. Details of spectral channels are given in Supplementary Table 2. For cell segmentation, brightfield images were used as a reference to create masks for the proximal tubule cells. Approximately 2–4 cells were selected from each image, and multiple multispectral images were taken for each patient.

### Cellular image analysis

After obtaining the spectral images, they were prepared for subsequent quantitative analysis using a multistep procedure to minimize sources of errors such as Poisson’s noise, dead or saturated pixels, background fluorescence and uneven illumination of the field of view, as reported in detail in^10,28^. Each multispectral image is pre-processed by using reference images (water, and calibration fluid images) as described in^27–29^. The uneven illumination of the field of view was then flattened, separately in each channel by using the image of the calibration fluid whose spectrum spans across all 34 spectral channels. Further image pre-processing steps comprised image equalization, primary denoising by removing undetectable pixels and outliers (spikes or dips)^27,28^. Furthermore, cells were manually segmented from the channel images to produce single-cell images^30^. During this segmentation process data register (image number, pixel coordinates, spectral channel etc.) were separately preserved for indexing the cell samples. Following image preparation and segmentation segmented single-cell images were used to extract the channel image features. These features include average channel intensity per cell, and associated measures such as channel intensity ratio, as defined in Reference^12^.

### Analysis of patient groups and statistics

Following the calculation of the cell image features, they were then scored to evaluate how well they differentiate the patient groups under consideration, and ranked using an independent evaluation criterion for binary classification (minimum attainable classification error)^31^ in Matlab 2019b. The best *n* cellular features were chosen for further analysis. This number of features, *n*, was kept lower than the number of cells in each group (*N*) to reduce the possibility of overfitting. To visualise the separation of different patient groups, the data points corresponding to *n*-dimensional feature vectors for each cell were first projected onto an optimal two-dimensional (2-D) space created by discriminative analysis^32,33^. This space was spanned by two canonical variables given by a specific linear combination of cellular features^34^. This space maximizes between-group distance while minimizing within-group variance^32^. We then developed a classifier to predict the pre-defined cell labels corresponding to the patients groups^35^, and the classifier performance was evaluated by using a ROC curve^35^. Nested cross validation was applied to validate our classifier for separating the groups which provides an unbiased performance assessment, by using external validation test^36^ (details are provided in Supplementary Material Section 1). The classified data have been additionally projected onto an optimally selected single dimension to produce maximally separated histograms. These data have also been visualised using boxplots. After confirming the non-parametric distribution of the data, the Mann–Whitney U test (two-tailed test) has been performed by using Matlab 2019b. Significant differences have been marked with *(for alpha < 0.05), **(for alpha < 0.01) and ***(for alpha < 0.001).

## Results

### Optimisation of cell storage condition

**Figure 1:**
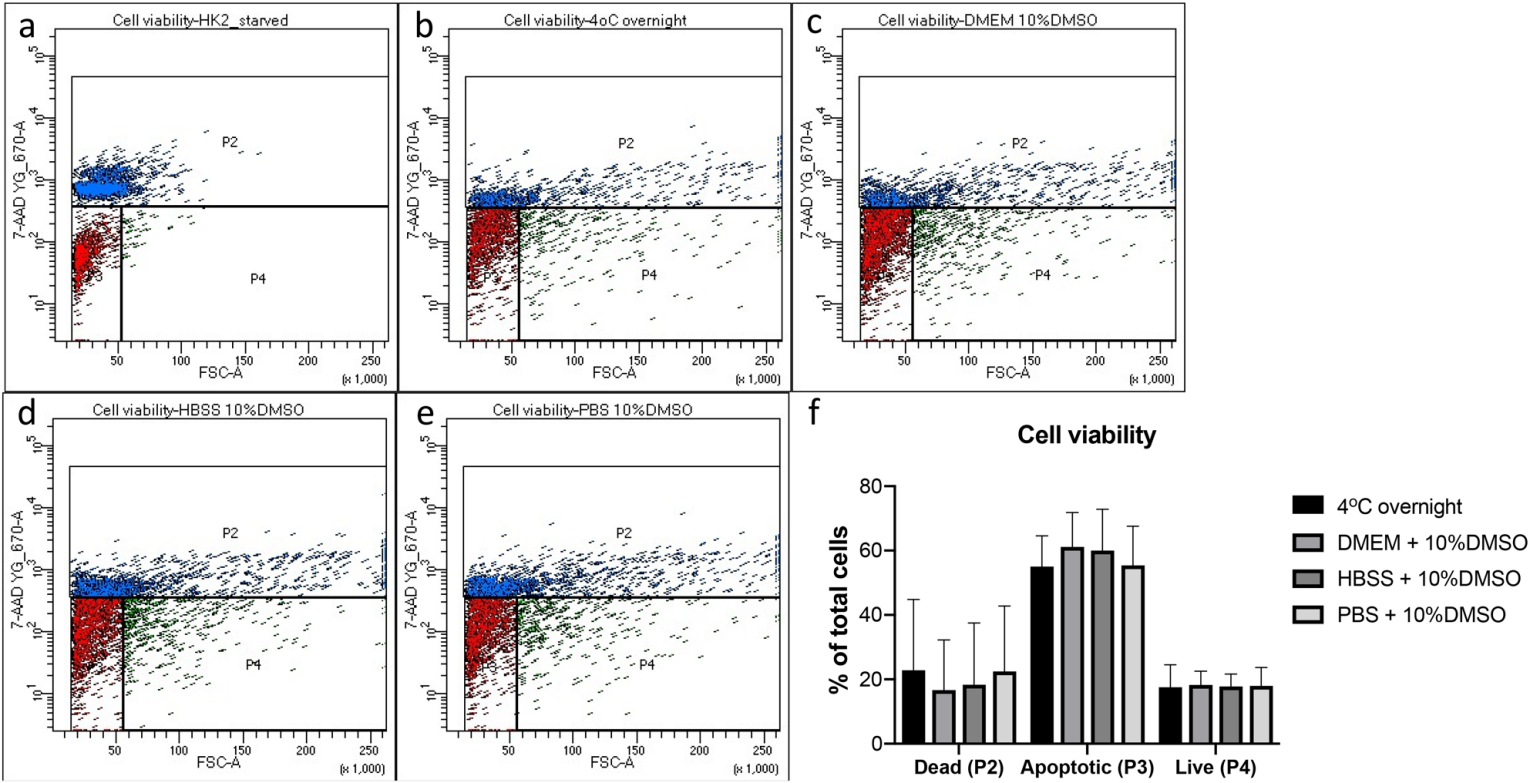
The effects of storage conditions on urinary cell viability. Cells are stored at different conditions and stained with 7-AAD. (**a**) Positive staining control: Starved HK2 cells; (**b**) Urinary cells kept at 4 °C overnight; (**c**–**e**) Urinary cells stored at − 80 °C in DMEM, HBSS and PBS + 10% DMSO respectively. (**f**) is the relative quantitation of **b**–**e**.

We have demonstrated that urinary exfoliated cells contain sufficient living cells (Fig. 1). Optimal storage conditions for cell viability were assessed and human kidney (HK2) cells were used as positive control. Our results demonstrated that either leaving the cells at 4 °C overnight or freezing them at − 80 °C in different buffers (DMEM, HBSS or PBS + 10% DMSO) led to similar levels of cell death and apoptosis.

**Figure 2:**
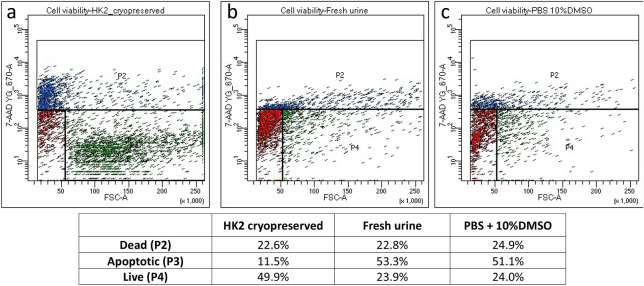
Cell viability. (**a**) HK2 cells cryopreserved in standard (DMEM + 10%DMSO); (**b**) Fresh urinary cells; (**c**) Urinary cells kept at − 80 °C in PBS + 10% DMSO.

We have additionally demonstrated that HK2 cells cryopreserved in the standard condition (DMEM + 10%DMSO) contained 22.6% dead cells, 11.5% apoptotic cells and nearly 50% live cells (Fig. 2). In comparison, urinary exfoliated cells are mostly composed of apoptotic cells (53.3%) and similar proportions of dead and live cells. Cryopreservation of urinary cells in PBS + 10%DMSO slightly increased the transition of apoptotic cells to dead cells yet maintained the same level of living cells, hence PBS was used for the rest of the experiment.

### Identification of PTCs in patients’ urine by CD13 and SGLT2 labelling

**Figure 3:**
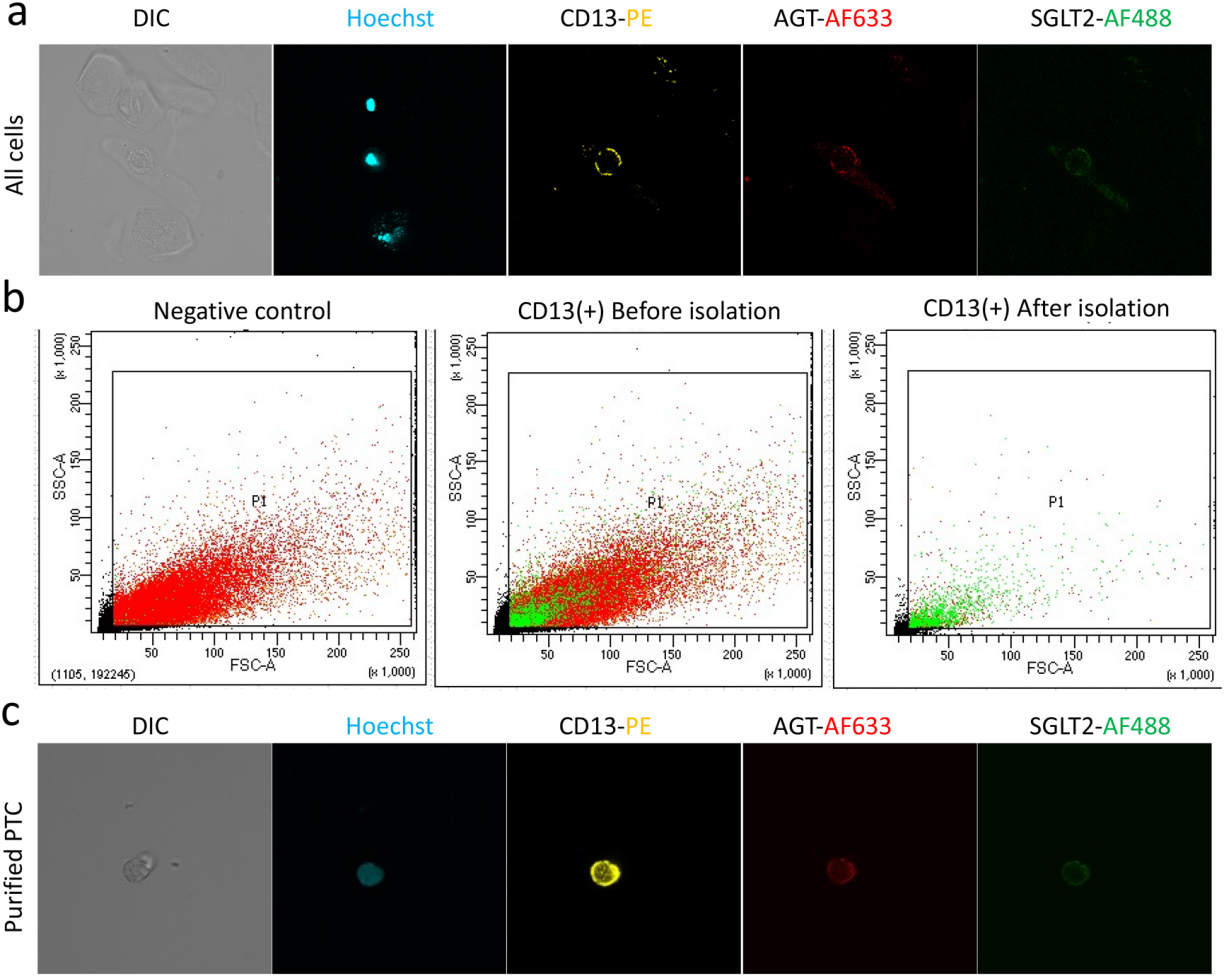
Urinary exfoliated cell characterisation before and after isolation (**a**) Immunofluorescence images showing only PTCs are positive for CD13, AGT and SGLT2; (**b**) Flow cytometry showing enrichment of CD13( +) cells before and after cell isolation; (**c**) Isolated cells have strong expression of PTC-specific markers.

The urine can contain different types of cells including podocytes, stem/progenitor cells, extracellular vesicles, distal cells, epithelial cells, bladder and collecting ducts cells^37^. Our result shows that CD13, Angiotensinogen (AGT) and SGLT2 are uniquely expressed on PTCs (as expected) and not in other epithelial cell types such as squamous and transitional epithelial cells that are present in the urine (Fig. 3a). Using flow cytometry, we demonstrated that sufficient numbers of CD13(+)/SGLT2(+) cells can be detected in the urine from different patients (Supplementary figure 1). According to supplementary figure 1, the majority of CD13-positive cells are also positive for SGLT2 but not necessarily vice versa. Kidney cells exfoliated into the urine from normal individuals and diabetic patients were labelled with CD13 and the proximal tubule marker SGLT2 then flow cytometry and confocal immunofluorescence analysis were carried out for cell characterisation. Our data demonstrated that isolated cells are not only enriched with CD13-positive cells (Fig. 3b) but also expressed specific markers of PTCs including AGT and SGLT2 (Fig. 3c).

**Figure 4:**
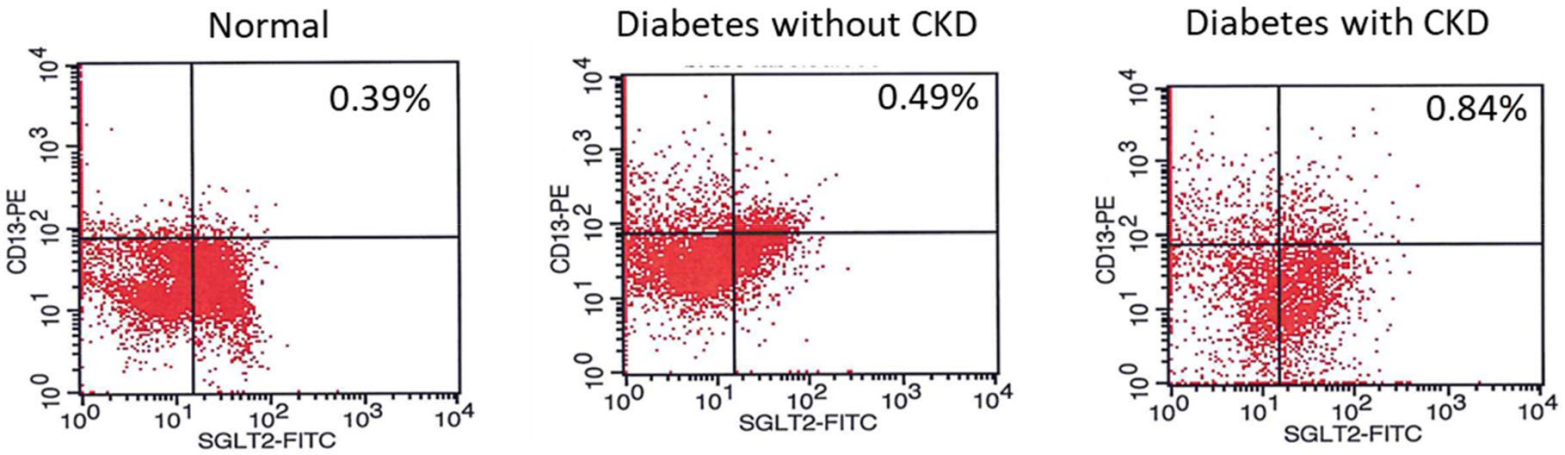
Flow cytometry showing that renal proximal tubule cells are present in the urine in normal subjects and diabetic patients with and without CKD. Human proximal tubule cells are in the right upper quadrant (positive for SLGT2 and CD13). The percentage of proximal tubule cells within total urinary exfoliated cells are shown, n = 3.

Our data showed that exfoliated renal PTCs (which are positive to CD13 and SGLT2) were detectable in normal individuals and diabetic patients with and without CKD (Fig. 4). This demonstrated the proof of concept that diabetic patients with and without CKD secrete viable exfoliated proximal tubule cells^38^ in the urine in sufficient amounts for further study.

### Differences in multispectral autofluorescence of exfoliated proximal tubule cells from diabetic patients with differing eGFR values

**Figure 5:**
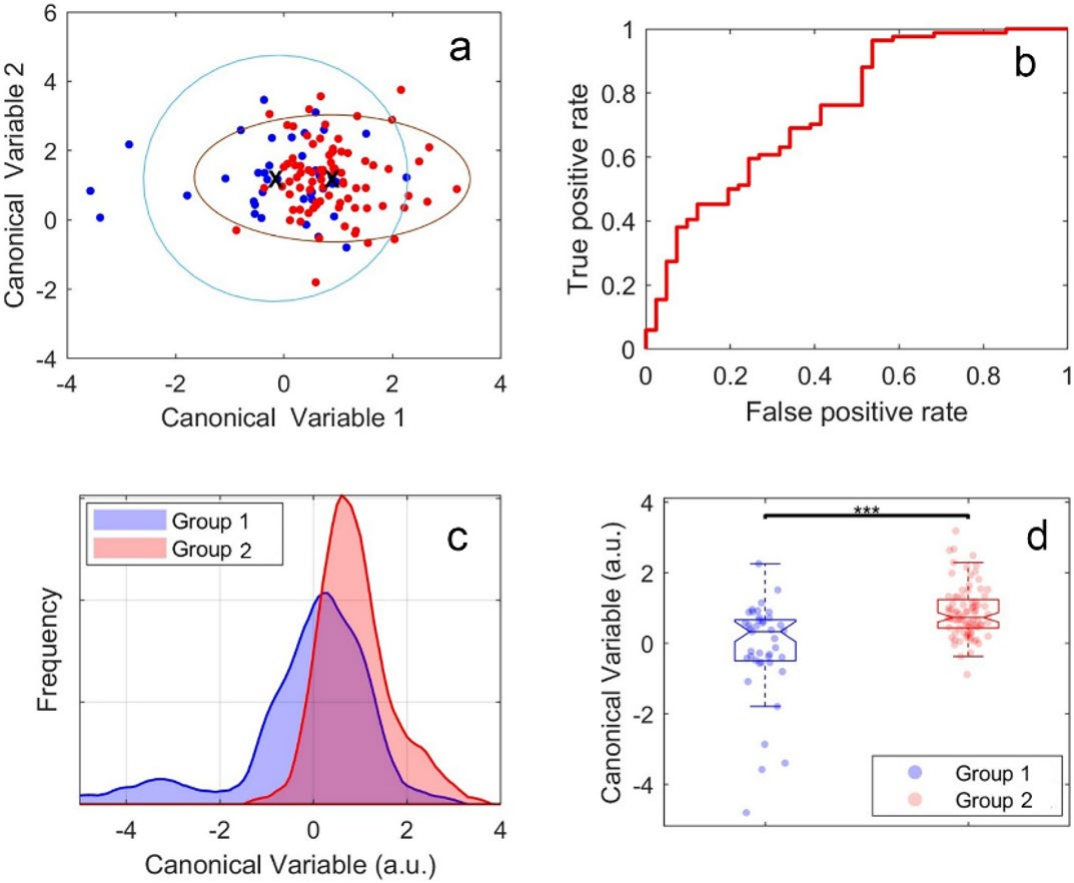
Differentiation of cells from patients with low and high eGFR levels by using six features selected from channel intensities and their ratios. (**a**) Clustering of cells in the two cell groups (group 1: n = 41 cells and group 2: n = 84 cells) under investigation. Symbols represent individual cells. (**b**) ROC curve for our obtained cell classifier (AUC = 0.75), (**c**) cell distribution histogram. (**d**) Boxplots corresponding to the cell classifier evaluated in (**b**). Symbols represent individual cells, *p* < 0.001.

**Figure 6:**
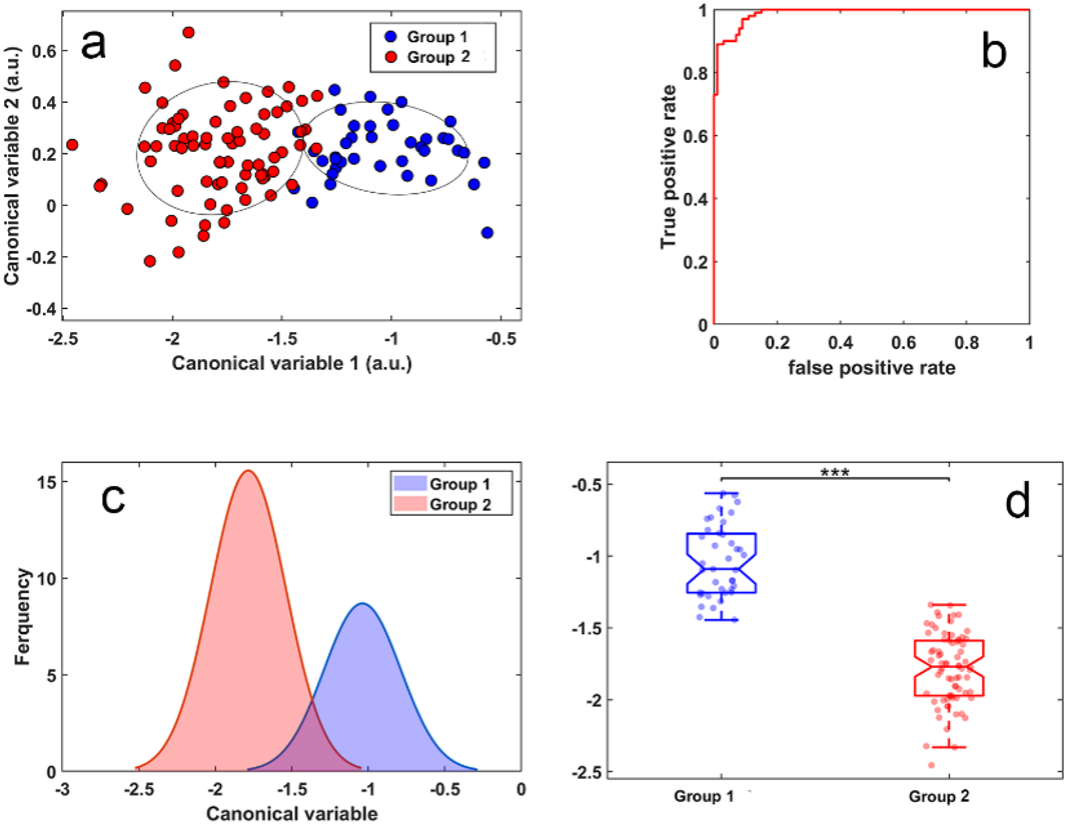
Differentiation of cells from patients with low and high eGFR levels using ten features. (**a**) Clustering of cells in the two cell groups (group1 and 2 has 41 cells and 84 cells respectively) under investigation. Symbols represent individual cells. (**b**) ROC curve for our obtained cell classifier (AUC = 0.99), (**c**) cell distribution histogram, (**d**) boxplots corresponding to the cell classifier evaluated in (**b**). Symbols represent individual cells, *p* < 0.001.

Urinary proximal tubule cells were extracted from the urine in the first cohort of diabetic patients with varying eGFR, indicating the presence of alterations in kidney function. Hyperspectral imaging and analysis performed as described in the Methods revealed significant differences in hyperspectral cellular feature patterns between cells from diabetic patients with eGFR > 60 ml/min/1.73 m^2^ (referred to as “high”) compared to those from patients with eGFR < 60 ml/min/1.73 m^2^ (referred to as “low”) as shown in Figs. 5 and 6. In Fig. 5 we have first limited our analysis to six cellular features selected only from among channel intensities and their ratios whose biological relevance can be identified. By using this approach, we were able to categorise cells with eGFR lower than 60 ml/min/1.73 m^2^ from eGFR > 60 ml/min/1.73 m^2^ with the area under the curve, AUC = 0.75 (Fig. 5a,b). Figure 5c shows the histograms for the datapoint shown in Fig. 5a produced by projection of data from Fig. 5a onto an optimised single direction. The same datapoints have been represented in boxplots in Fig. 5d which shows statistical differences between both groups of cells. The features applied in this conservative model (Fig. 5) are listed in Supplementary Table 3.

To explore the potential upper limit of multispectral analysis' ability to classify cells into low eGFR (< 60 ml/min/1.73 m^2^) and high eGFR (> 60 ml/min/1.73 m^2^), a second analysis was performed where we used up to ten features and we included features which do not currently have a biologically interpretable meaning. This resulted in excellent separation of these two classes of cells and an AUC of 0.99 (Fig. 6a,b). The corresponding histograms and boxplots are shown in Fig. 6c,d. Features applied in this expanded model (Fig. 6) are detailed in Supplementary Table 4.

### Differences in multispectral autofluorescence of exfoliated proximal tubule cells from patients without and with different stages of renal structural pathology

**Figure 7:**
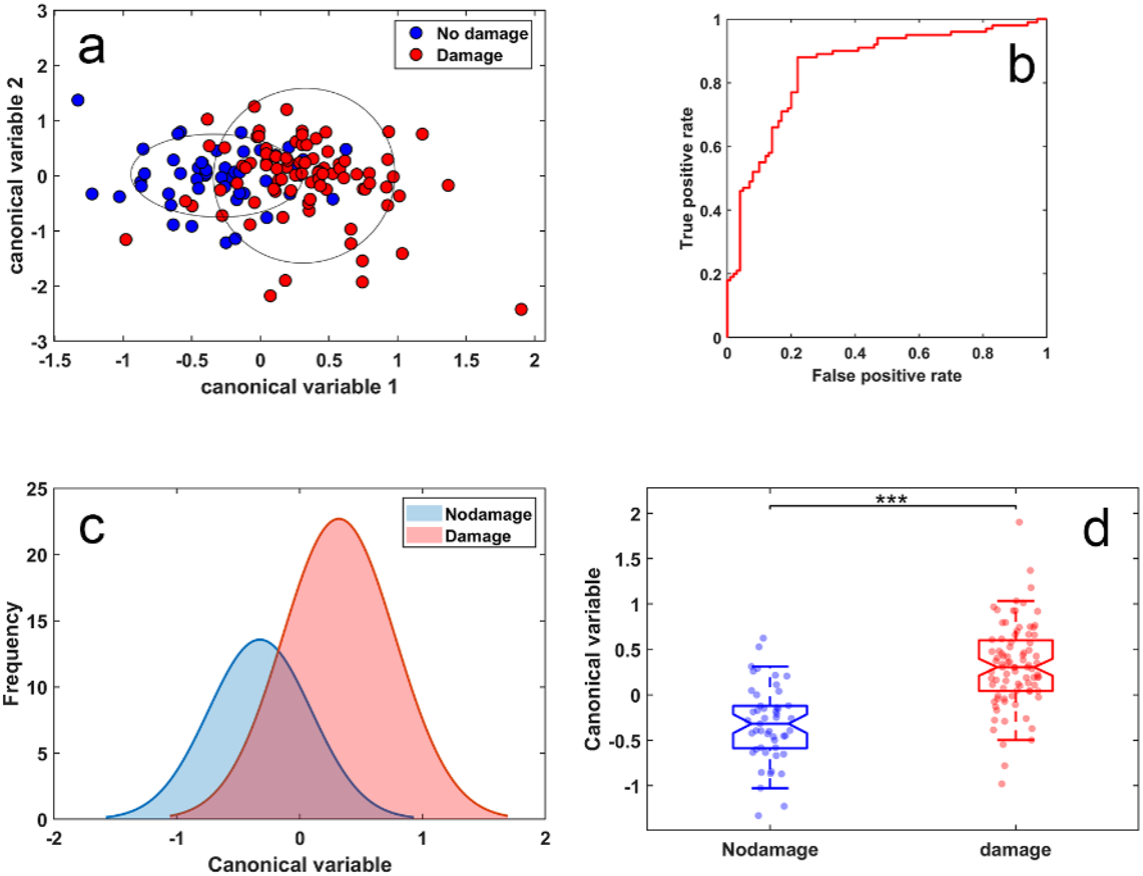
Correlation between multispectral changes in proximal tubule cells from patients with and without renal pathology. (**a**) Cluster formed using six feature analysis to separate cells from patients with no observed fibrosis (n = 41 cells) from those with any fibrosis (mild and moderate/severe, n = 95 cells). Symbols represent individual cells.; (**b**) corresponding ROC analysis indicating AUC = 0.85. (**c**) Cell distribution histogram, (**d**) boxplots corresponding to the cell classifier evaluated in (**b**). Symbols represent individual cells, *p* < 0.001.

**Figure 8:**
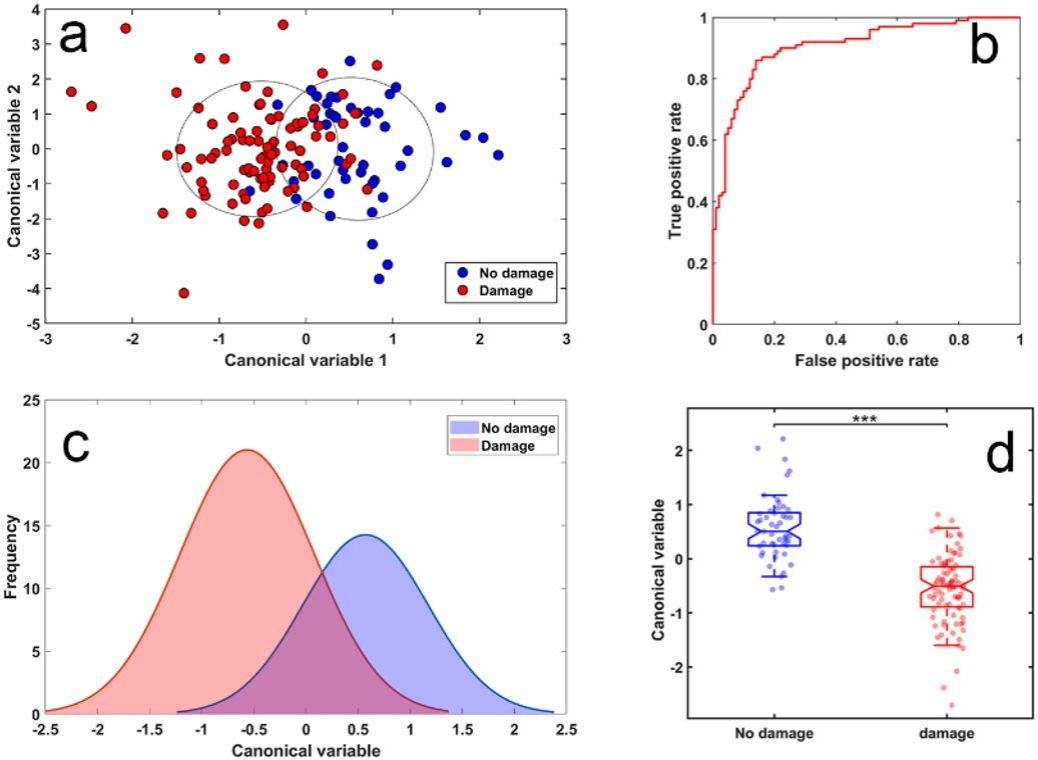
Correlation between multispectral changes in proximal tubule cells from patients with and without renal pathology. (**a**) Cluster formed with 8 features to separate cells from patients with no observed fibrosis (n = 41 cells) from those with fibrosis (n = 95 cells). Symbols represent individual cells. (**b**) ROC curve for our obtained cell classifier (AUC = 0.90). (**c**) Cell distribution histogram. (**d**) Boxplots corresponding to the cell classifier evaluated in (**b**). Symbols represent individual cells, *p* < 0.001.

Kidney fibrosis in patients’ cohort 2 was directly assessed through pathological examination of kidney biopsies. In order to detect the corresponding changes in multispectral autofluorescence images, we employed a six feature model with features limited to channel intensities and their ratios, similar to those used for the classification of patients with respect to their value of eGFR previously shown in Fig. 5. We found that cells from patients with no observed renal fibrosis could be separated from those with any renal fibrosis with AUC = 0.85 (Figs. 7a,b). The corresponding histogram and boxplots are shown in Fig. 7c,d. The second, alternative analysis with an extended set of 8 features (Fig. 8) achieved the AUC of 0.90. The spectral features used are listed in Supplementary Table 5 for the conservative model from Fig. 7 and Supplementary Table 6 for the extended model from Fig. 8.

## Discussion

Cells represent a rich source of information for monitoring dynamic processes in the living body. While circulating cells are already widely used for cancer diagnostics, the analysis of renal cells exfoliated into the urine has the potential to provide a novel window into the physiology of otherwise inaccessible organs such as kidneys. The data presented here focuses on renal health, but the approach to diagnose exfoliated cells is likely to extend to other targets. This is the first time that detailed high-content imaging has been systematically applied to kidney cells in an attempt to probe the cellular morphology of autofluorescence in this chronic disease. Multispectral characteristics of autofluorescence has the advantage that single exfoliated cells can be analysed; this is important as the number of kidney cells which can be extracted from urine may be low.

In this work we have developed a long-term cryopreservation protocol for urinary exfoliated cells. We also successfully isolated human PTCs from the urine using CD13 and SGLT2 antibodies as previously demonstrated^25^. SGLT2 is known to be expressed in the S1 segment of human PTCs^39,40^. We have also additionally demonstrated that sufficient PTCs are exfoliated in the urine from patients with healthy or diseased kidneys. We also explored whether eGFR changes, and, separately, kidney tissue pathology changes, both related to kidney disease have the potential to be reflected in autofluorescence properties of kidney cells exfoliated in the urine. Such in-depth analysis is important in CKD because the alternative approach of counting urinary kidney cells does not always correspond to the degree of kidney dysfunction^5^. Autofluorescence sensitively reflects cellular processes, most notably metabolism^11,15^, but also physiologically significant measures such as cellular levels of reactive oxygen species^16^. Cell conditions which are as physiologically authentic as possible allow more accurate autofluorescence diagnostics. However, in clinical settings, it is not always feasible for samples to be assessed immediately, and long-term storage is usually preferred. Inappropriate storage and shipment conditions could impair cellular viability and otherwise alter cells, thus it is important to ensure temperature and buffer for storage are optimised. In this study we determined the optimal conditions for the extraction of urinary exfoliated proximal tubule cells for multispectral assessment of cell autofluorescence.

We then isolated cells from the urine of patients with diabetes mellitus with different levels of eGFR indicating varying degrees of kidney dysfunction and from patients from whom kidney biopsies had been taken and assessed for fibrosis (the gold standard for diagnosis of kidney dysfunction). We were successful in categorising these cells using their autofluorescent characteristics assessed through multispectral microscopy and related these characteristics to a binary kidney health status based on eGFR values and, separately, on kidney pathology determined from biopsies. This was explored in two models: a more conservative model with a smaller number of cell features drawn from channel intensities and their ratios, and an extended model where more features and also additional types of mathematically abstract features were allowed (e.g. kurtosis). Limiting the features used to channel intensities and ratios allows the features to be more closely related to biological properties such as the content of individual fluorophores, some of whom tend to dominate specific spectral channels (for example intensity in channel 2 is predominately related to NAD(P)H content and intensity in channels 15 and 32 is dominated by flavins^11,41^. The contribution of pure fluorophores to specific channels may be further exacerbated by thresholding the signal and taking top 10% of the pixels, as this may reflect localisation of such fluorophores in specific cellular compartments such as mitochondria. Similarly, the ratio of intensities of some channels (e.g. channel 2/channel 32) is related to the cellular redox ratio^11,41^. Thus, by using the conservative model we increased the likelihood of the observed changes to be underpinned by defined and known biological changes in cells as kidney dysfunction develops.

Low sample size limited the complexity of the models we were able to apply due to the risks of overfitting, however in secondary analyses, when the restrictions on the models were loosened, very high accuracy was achieved. Greater accuracy was achieved in a cohort where cells were assessed relative to the status of kidney biopsies. This is not surprising as the proximal tubule cells which we examined by multispectral assessment originated directly from the kidney cells and their characteristics should directly reflect the biological status of the kidneys, more so than a secondary measure like eGFR. A further limitation of this study is that we are unable to relate any changes in autofluorescence to other potential factors of influence, limiting any causal relationships which could be determined based on our data. Putative parameters potentially affecting cell autofluorescence may include age (through cell senescence) and sex, while the potential clinical factors may include eGFR, urinary protein, HbA1c etc. Among these factors, age and sex were matched between first and second cohort, while the levels of eGFR were independently analysed. We are mindful that the difference in eGFR in the second cohort could have an impact on autofluorescence instead of fibrosis. A larger cohort is required in order to match eGFR levels in for cohort 2 to confirm whether the levels of renal fibrosis are responsible for changes in hyperspectral autofluorescence independent of eGFR levels. Taken together, our findings indicate that multispectral measurement of proximal tubule cell autofluorescence—autofluorescence colour—is different in healthy and diseased kidneys.

## Materials and correspondence

The data that support the findings of this study are available from the corresponding author on request.

## Supplementary Information

Supplementary Information.

## References

[1] Zhang JL, Rusinek H, Chandarana H, Lee VS (2013). Functional MRI of the kidneys. J. Magn. Reson Imaging.

[2] Sharma K (2017). Kidney volume measurement methods for clinical studies on autosomal dominant polycystic kidney disease. PLoS ONE.

[3] Kitajima K (2016). Update on advances in molecular PET in urological oncology. Jpn. J. Radiol..

[4] Dekel B, Reisner Y (2004). Engraftment of human early kidney precursors. Transpl. Immunol..

[5] Oliveira Arcolino F (2015). Human urine as a noninvasive source of kidney cells. Stem Cells Int..

[6] Mackensen-Haen S (1992). The consequences for renal function of widening of the interstitium and changes in the tubular epithelium of the renal cortex and outer medulla in various renal diseases. Clin. Nephrol..

[7] Eddy AA (2014). Overview of the cellular and molecular basis of kidney fibrosis. Kidney Int. Suppl..

[8] Dorrenhaus A (2000). Cultures of exfoliated epithelial cells from different locations of the human urinary tract and the renal tubular system. Arch. Toxicol..

[9] Inoue CN (2003). Reconstruction of tubular structures in three-dimensional collagen gel culture using proximal tubular epithelial cells voided in human urine. vitro Cell. Dev. Biol. Anim..

[10] Habibalahi, A., Bala, C., Allende, A., Anwer, A. G. & Goldys, E. M. Novel automated non invasive detection of ocular surface squamous neoplasia using multispectral autofluorescence imaging. *Ocular Surf.* **17**, 540–550 (2019). 10.1016/j.jtos.2019.03.003 30904597

[11] Gosnell, M. E., Anwer, A. G., Cassano, J. C., Sue, C. M. & Goldys, E. M. Functional hyperspectral imaging captures subtle details of cell metabolism in olfactory neurosphere cells, disease-specific models of neurodegenerative disorders. *Biochimica et Biophysica Acta (BBA)-Mol. Cell Res.* **1863**, 56–63 (2016). 10.1016/j.bbamcr.2015.09.030 26431992

[12] Gosnell ME (2016). Quantitative non-invasive cell characterisation and discrimination based on multispectral autofluorescence features. Sci. Rep..

[13] El Aziz MA, Selim IM, Xiong S (2017). Automatic detection of galaxy type from datasets of galaxies image based on image retrieval approach. Sci. Rep..

[14] Campbell JM (2019). Non-destructive, label free identification of cell cycle phase in cancer cells by multispectral microscopy of autofluorescence. BMC Cancer.

[15] Mahbub SB (2019). Non-invasive monitoring of functional state of articular cartilage tissue with label-free unsupervised hyperspectral imaging. Sci. Rep..

[16] Habibalahi, A. *et al.* Non-invasive real-time imaging of reactive oxygen species (ROS) using multispectral auto-fluorescence imaging technique: a novel tool for redox biology. *Redox Biol.* **34**, 101561 (2020). 10.1016/j.redox.2020.101561 PMC7287272 32526699

[17] Habibalahi A (2019). Optimized autofluorescence spectral signature for non-invasive diagnostics of ocular surface squamous neoplasia (OSSN). IEEE Access.

[18] Bertoldo MJ (2020). NAD(+) repletion rescues female fertility during reproductive aging. Cell Rep..

[19] Glastras SJ (2016). Effect of GLP-1 receptor activation on offspring kidney health in a rat model of maternal obesity. Sci. Rep..

[20] Stangenberg S (2015). Oxidative stress, mitochondrial perturbations and fetal programming of renal disease induced by maternal smoking. Int. J. Biochem. Cell Biol..

[21] Granata S (2009). Mitochondrial dysregulation and oxidative stress in patients with chronic kidney disease. BMC Genomics.

[22] Modaresi A, Nafar M, Sahraei Z (2015). Oxidative stress in chronic kidney disease. Iran. J. Kidney Dis..

[23] Brachemi S, Bollée G (2014). Renal biopsy practice: What is the gold standard?. World. J. Nephrol..

[24] Gill AJ (2014). Succinate dehydrogenase (SDH)-deficient renal carcinoma: a morphologically distinct entity: a clinicopathologic series of 36 tumors from 27 patients. Am. J. Surg. Pathol..

[25] Rahmoune H (2005). Glucose transporters in human renal proximal tubular cells isolated from the urine of patients with non–insulin-dependent diabetes. Diabetes.

[26] Juarez J, Bradstock KF, Gottlieb DJ, Bendall LJ (2003). Effects of inhibitors of the chemokine receptor CXCR4 on acute lymphoblastic leukemia cells in vitro. Leukemia.

[27] Rehman AU (2017). Fluorescence quenching of free and bound NADH in HeLa cells determined by hyperspectral imaging and unmixing of cell autofluorescence. Biomed. Opt. Express.

[28] Mahbub SB, Plöschner M, Gosnell ME, Anwer AG, Goldys EM (2017). Statistically strong label-free quantitative identification of native fluorophores in a biological sample. Sci. Rep..

[29] Mahbub, S. B. *Unsupervised Hyperspectral Unmixing Analysis for Label-free Quantitative Identification of Native Fluorophores in a Biological Sample by a Robust Dependent Component Analysis (RoDECA)*, Macquarie University, Faculty of Science and Engineering, Department of Science and Engineering, Department of Physics and Astronomy (2017).

[30] Wang D, Bodovitz S (2010). Single cell analysis: the new frontier in ‘omics’. Trends Biotechnol..

[31] Reyes-Aldasoro CC, Bhalerao A (2006). The Bhattacharyya space for feature selection and its application to texture segmentation. Pattern Recogn..

[32] Jombart T, Devillard S, Balloux F (2010). Discriminant analysis of principal components: a new method for the analysis of genetically structured populations. BMC Genet..

[33] Johnson RA, Wichern DW (2002). Applied Multivariate Statistical Analysis.

[34] Naganathan GK (2008). Visible/near-infrared hyperspectral imaging for beef tenderness prediction. Comput. Electron. Agric..

[35] Vapnik V (2013). The Nature of Statistical Learning Theory.

[36] Vabalas A, Gowen E, Poliakoff E, Casson AJ (2019). Machine learning algorithm validation with a limited sample size. PLoS ONE.

[37] Oliveira Arcolino, F. *et al.* Human urine as a noninvasive source of kidney cells. *Stem Cells Int.* **2015**, 362562 (2015). 10.1155/2015/362562 PMC4451513 26089913

[38] Detrisac C, Mayfield R, Colwell J, Garvin A, Sens D (1983). In vitro culture of cells exfoliated in the urine by patients with diabetes mellitus. J. Clin. Invest..

[39] Kamiyama M, Garner MK, Farragut KM, Kobori H (2012). The establishment of a primary culture system of proximal tubule segments using specific markers from normal mouse kidneys. Int. J. Mol. Sci..

[40] Kamiyama M (2007). Polymorphisms in the 3′ UTR in the neurocalcin δ gene affect mRNA stability, and confer susceptibility to diabetic nephropathy. Hum. Genet..

[41] Reyes JMG (2006). Metabolic changes in mesenchymal stem cells in osteogenic medium measured by autofluorescence spectroscopy. Stem Cells.

