# Publisher Correction: Non-invasive assessment of exfoliated kidney cells extracted from urine using multispectral autofluorescence features

**DOI:** 10.1038/s41598-021-96178-x

**Published:** 2021-09-15

**Authors:** Saabah B. Mahbub, Long T. Nguyen, Abbas Habibalahi, Jared M. Campbell, Ayad G. Anwer, Uzair M. Qadri, Anthony Gill, Angela Chou, Muh Geot Wong, Martin E. Gosnell, Carol A. Pollock, Sonia Saad, Ewa M. Goldys

**Affiliations:** 1 ARC Centre of Excellence for Nanoscale Biophotonics, UNSW Sydney, Sydney, NSW 2052 Australia; 2 Graduate School of Biomedical Engineering, UNSW Sydney, Sydney, NSW 2052 Australia; 3 School of Mathematical and Physical Sciences, Faculty of Science, University of Technology, Sydney, Australia; 4 Kolling Institute of Medical Research, Royal North Shore Hospital, St Leonards, NSW 2065 Australia; 5 NSW Health Pathology, Department of Anatomical Pathology, Royal North Shore Hospital, Sydney, NSW 2065 Australia; 6 Sydney Medical School, University of Sydney, Sydney, NSW 2006 Australia; 7 The George Institute for Global Health, Sydney, NSW 2050 Australia; 8 Quantitative Pty Ltd, 118 Great Western Highway, Mount Victoria, NSW 2786 Australia

Correction to: *Scientific Reports* 10.1038/s41598-021-89758-4, published online 20 May 2021

In the original version of this Article Saabah B. Mahbub and Long T. Nguyen were omitted as equally contributing authors.

Additionally, Sonia Saad and Ewa M. Goldys were omitted as jointly supervised authors.

This error has now been corrected in the PDF version of the Article; the HTML version was correct from the time of publication.

