# Supplementary material for: Non-invasive assessment of exfoliated kidney cells extracted from urine using multispectral autofluorescence features
Source: Sci Rep. 2021 May 20;11:10655. doi: 10.1038/s41598-021-89758-4 (PMC8138006; doi:10.1038/s41598-021-89758-4)
Supplement: Supplementary file 1 — Supplementary Information. [file 41598_2021_89758_MOESM1_ESM.pdf]

Supplementary Material: Non-invasive assessment of exfoliated kidney cells extracted from urine using multispectral autofluorescence features

Saabah B. Mahbub[1,2*#], Long T. Nguyen[5*], Abbas Habibalahi[1,2], Jared M. Campbell[1,2], Ayad G. Anwer[1,2], Uzair M. Qadri[3], Anthony Gill[4,5,6], Angela Chou[4,5,6], Muh Geot Wong[7], Martin E. Gosnell[8], Carol A. Pollock[4,6], Sonia Saad[4,6#ǂ], Ewa M. Goldys[1,2#ǂ]

[1] ARC Centre of Excellence for Nanoscale Biophotonics, UNSW Sydney, NSW 2052, Australia.
[2] Graduate School of Biomedical Engineering, UNSW Sydney, NSW 2052, Australia.
[3] School of Clinical and Molecular Biosciences, Faculty of Science, Centre for Health Technology, University of Technology, Sydney, Australia.
[4] Kolling Institute of Medical Research, Royal North Shore Hospital, St Leonards NSW 2065, Australia.
[5] NSW Health Pathology, Department of Anatomical Pathology, Royal North Shore Hospital, Sydney NSW 2065 Australia.
[6] Sydney Medical School, University of Sydney, Sydney, NSW 2006, Australia.
[7] The George Institute for Global Health, NSW 2050, Australia.
[8] Quantitative Pty Ltd, 118 Great Western Highway, Mount Victoria, NSW 2786, Australia.

***,** ǂ These authors contributed equally
# Corresponding Author's email: s.mahbub@unsw.edu.au; sonia.saad@sydney.edu.au; e.goldys@unsw.edu.au

Supplementary Table

Supplementary Table 1(a): e-GFR data for Patients from Cohort 1. Blue – Group 1 (eGFR > 60), Red- Group 2 (eGFR < 60)

Group	Exp ID	eGFR
Group 1	P13	103
	P17	103
	P20	102
	P9	63
	P11	78
	P14	90
	P19	81
	P21	92
	P22	62
	P27	74
Group 2	P12	55
	P15	36
	P16	37
	P18	42
	P25	43
	P26	43
	P29	50
	P31	35
	P23	9

	P24	12
	P8	22
	P10	10
	P28	28
	P30	30

Supplementary Table 1(b): Patients from Cohort 2- without and with renal pathology.

Group	Exp ID	Interstitial fibrosis score	Diabetic
No damage	B6	0	Yes
	B25	0	No
	B26	0	No
Damage	B5	1	No
	B15	1	No
	B22	1	Yes
	B28	1	No
	B23	2	No
	B24	3	No
	B29	3	No

Supplementary Table 2 Details of spectral channels used in multispectral imaging

Spectral Channels Number	Excitation wavelength (bandwidth) (nm)	Emission wavelength (bandwidth) (nm)	Dichroic Mirror longpass (nm)	Exposure (sec)	EM Gain	No of image accumulations to calculate “averages “	Power at Objective (µW)
1	345	414	389	5	1	3	2.8
2	345	451	389	5	1	3	2.9
3	345	575	552	5	1	3	2.7
4	490	575	552	5	1	3	4.0
5	505	575	552	5	1	3	8.4
6	345	594	552	5	1	3	2.9
7	490	594	552	5	1	3	4.1
8	505	594	552	5	1	3	8.6
9	358	414	389	5	1	3	2.8

10	371	414	389	5	1	3	3.1
11	377	414	389	5	1	3	2.1
12	371	451	389	5	1	3	3.1
13	377	451	389	5	1	3	2.2
14	381	451	389	5	1	3	0.9
15	358	575	552	5	1	3	3.0
16	371	575	552	5	1	3	4.6
17	377	575	552	5	1	3	8.5
18	381	575	552	5	1	3	10.2
19	391	575	552	5	1	3	6.9
20	397	575	552	5	1	3	8.5
21	400	575	552	5	1	3	9.2
22	403	575	552	5	1	3	4.9
23	406	575	552	5	1	3	9.7
24	412	575	552	5	1	3	13.5
25	437	575	552	5	1	3	14.3
26	457	575	552	5	1	3	8.3
27	406	594	552	5	1	3	10.2
28	412	594	552	5	1	3	14.3
29	418	594	552	5	1	3	15.5
30	430	594	552	5	1	3	10.7
31	437	594	552	5	1	3	14.9
32	457	594	552	5	1	3	8.6
33	469	594	552	5	1	3	12.3
34	476	594	552	5	1	3	13.4
35	476	575	552	0.01	1	1	

Supplementary Table 3: Spectral features used to differentiate high eGFR (Group 1) from low eGFR (Group 2); conservative model. Patients are from Cohort 1 and data are shown in Figure 5.

No	Feature description
1	Ratio of mean value of top 10% of channel 18 and mean value of top 10% of channel 17
2	Ratio of mean value channel 19 and mean value of channel 17
3	Ratio of mean value of top 10% of channel 9 and mean value of channel 28
4	Ratio of mean value of top 10% of channel 19 and mean value of top 10% of channel 17
5	Ratio of mean value of channel 1 and mean value of top 10% of channel 2
6	Ratio of mean value of top 10% of channel 27 and mean value of top 10% of channel 1

Supplementary Table 4: Spectral features used to differentiate Group 1 from Group 2; expanded model. Patients are from Cohort 1, and data are shown in Figure 6.

No	Feature description	No	Feature description
1	Mean value channel 7 divided by mean value of top 40% of channel 30	6	Mean value channel 32 divided by mean value of channel 28
2	Mean value channel 25 divided by mean value of top 40% of channel 3	7	Mean value channel 22 divided by mean value of top 40% of channel 10
3	Mean value channel 13 divided by mean value of principal component 3	8	Mean value channel 8 times by mean value of channel 22
4	Mean value of channel 2 divided by mean value of top 40% of channel 13	9	Mean value channel 19 divided by mean value of top 40% of channel 5
5	Skewness of top 40% of channel 26	10	Mean value channel 14 times by mean value of channel 17

Supplementary Table 5. Spectral features used to differentiate cells from patients with no observable renal pathology from other patients, conservative model. Patients are from Cohort 2 and data are shown in Figure 7.

No	Feature description	No	Feature description
1	Mean value channel 4 divided by mean value of channel 24	4	Mean value channel 23 divided by mean value of top 40% of channel 18
2	Mean value channel 20 divided by mean value of channel 28	5	Mean value channel 36 divided by mean value of channel 28
3	Mean value channel 5 divided by mean value of top 40% of channel 21	6	Mean value channel 7 divided by mean value of channel 16

Supplementary Table 6. Spectral features used to differentiate cells from patients with no observable renal pathology from other patients, conservative model. Patients are from Cohort 2 and data are shown in Figure 8.

No	Feature description	No	Feature description
1	Mean value channel 23 divided by mean value of top 40% of channel 18	5	Variance of top 40% of channel 1
2	Kurtosis of channel 13	6	Skewness of top 40% of channel 9
3	Mean value channel 5divided by mean value of top 40% of channel 21	7	Mean value channel 36 divided by mean value of top 40% of channel 30
4	Mean value channel 4 divided by mean value of top 40% of channel 24	8	Mean value channel 7 divided by mean value of top 40% of channel 16

Supplementary Material Section 1:

We have demonstrated that urinary exfoliated cells express detectable amounts of renal proximal tubular cells that are positively for both CD13 and SGLT2 (top right quadrant, Supplementary Figure 1).

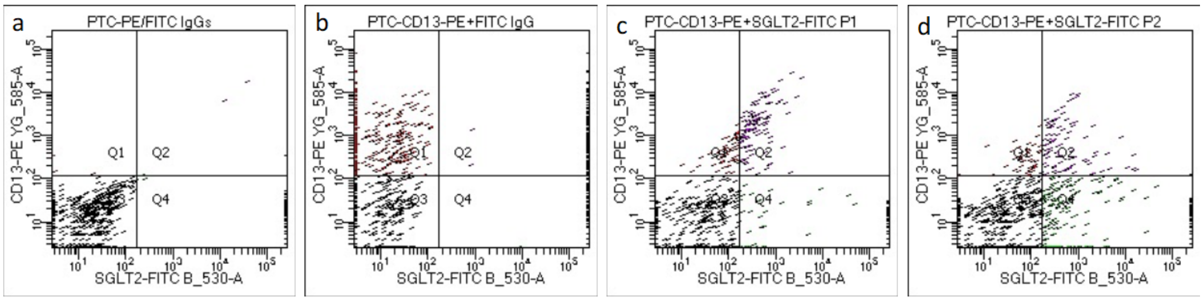

Supplementary Figure 1. Urinary cells stained with (a) PE and FITC IgG controls, (b) CD13-PE and FITC-IgG control, and (c, d) CD13-PE and SLGT2-FITC. (b) and (c) are from two different urine sample.

Supplementary Material Section 2:

To validate our classifier for separating the groups (i.e. based on eGFR in **Section 3.3** or based on renal pathology in **Section 3.4**), we used nested cross validation which provides an unbiased performance assessment using external validation test[1]. Here training dataset (80% of data) used to create the discriminative space and the remaining 20% data formed the testing dataset for discriminative space evaluation. This process was repeated 5 times by dividing data set for 5 different subsets[2]. For each step, training dataset are projected onto an optimal two-dimensional (2-D) space created by discriminative analysis[3,4] and formed two clearly separate clusters of Group 1 (Blue circle data points) and Group 2 (Red circle data points), shown in Supplementary Figure 2 a. Next, testing dataset (Group 1 with Blue crosses, and Group 2 with Red crosses) was reflected to the same space followed by calculating the classifier accuracy. This is repeated five times to use all five subsets and average accuracy for cohort 1 (based on eGFR in Section 3.3) is 76.25±9.4%. In a similar way the calculated accuracy value for cohort 2 (shown in Supplementary Figure 2 b) is 79.96±1.4%.

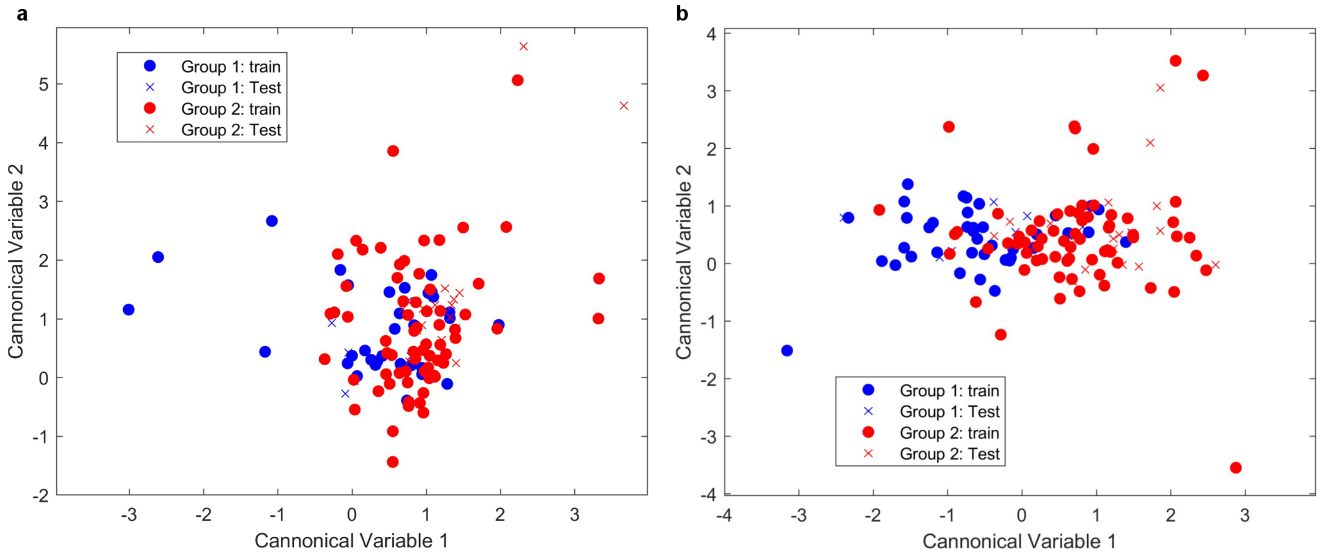

Supplementary Figure 2. (a) Classifier validation through testing and training cross-validation process with varying levels of (a) eGFR (cohort 1, **Supplementary Table 1 a**) and (b) renal pathology (cohort 2, **Supplementary Table 1 b**) by using six features. Symbols represent individual cells from each group.

Supplementary Material Section 3:

Bright field and spectral channel images from the representative cell from each group are presented in **Supplementary Figure 3**. First and second row presents the cell from Group 1 (eGFR>60) and Group 2 (eGFR<60) respectively from **Section 3.3**. Here first column presents the brightfield image, and next three columns represents

the autofluorescence signal from the channel number 1, 18 and 28 to show the spectral difference between the groups under investigation.

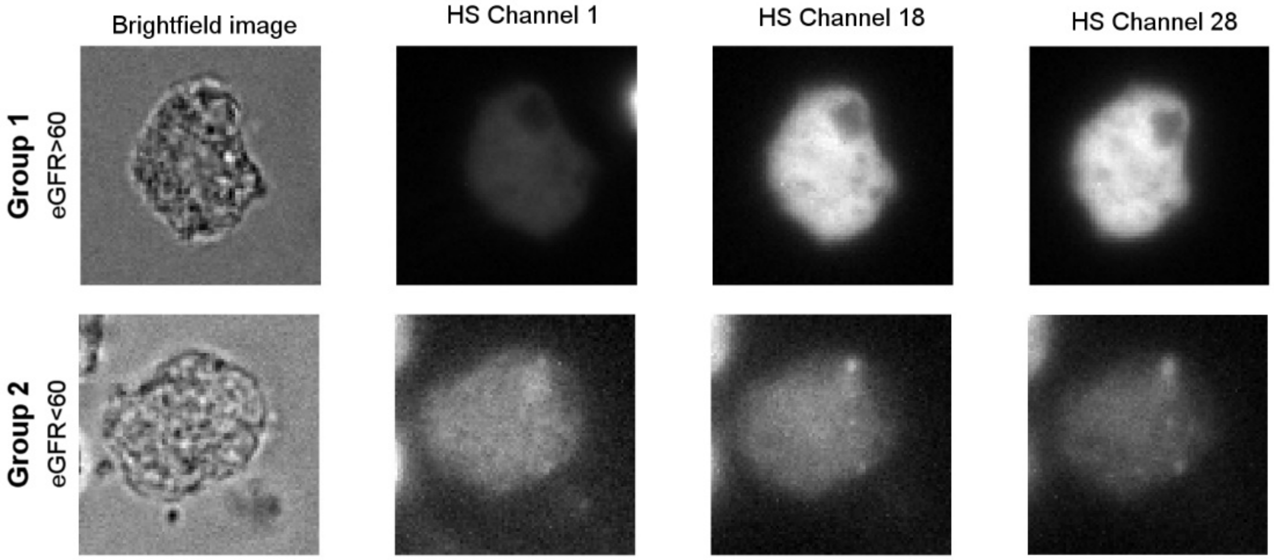

Supplementary Figure 3. Bright field and spectral channel images from the representative cell from cohort 1 (**Section 3.3/ Supplementary Table 1a**). Contrast of the HS channel images are rescaled for visualization purpose only.

References:

1 Vabalas, A., Gowen, E., Poliakoff, E. & Casson, A. J. Machine learning algorithm validation with a limited sample size. *PloS one* **14**, e0224365 (2019).

2 Habibalahi, A., Bala, C., Allende, A., Anwer, A. G. & Goldys, E. M. J. T. o. s. Novel automated non invasive detection of ocular surface squamous neoplasia using multispectral autofluorescence imaging. (2019).

3 Jombart, T., Devillard, S. & Balloux, F. Discriminant analysis of principal components: a new method for the analysis of genetically structured populations. *BMC genetics* **11**, 94 1471-2156 (2010).

4 Johnson, R. A. & Wichern, D. W. *Applied multivariate statistical analysis*. Vol. 5 (Prentice hall Upper Saddle River, NJ, 2002).
